# Two Novel Myoviruses from the North of Iraq Reveal Insights into *Clostridium difficile* Phage Diversity and Biology

**DOI:** 10.3390/v8110310

**Published:** 2016-11-16

**Authors:** Srwa J. Rashid, Jakub Barylski, Katherine R. Hargreaves, Andrew A. Millard, Gurinder K. Vinner, Martha R. J. Clokie

**Affiliations:** 1 Department of Infection, Immunity and Inflammation, Medical Sciences Building, University of Leicester, University Road, Leicester, LE1 9HN, UK; saroa.rashid@gmail.com; 2 Department of Molecular Virology, Faculty of Biology, Adam Mickiewicz University in Poznan, Poznan 61-712, Poland; erytropoeta@gmail.com; 3 Department of Microbiology, The Ohio State University, Columbus, OH 43201, USA; krhargr@gmail.com; 4 Microbiology & Infection Unit, Warwick Medical School, University of Warwick, Coventry, CV4 7AL, UK; andrew.millard@warwick.ac.uk; 5 Department of Chemical Engineering, Loughborough University, Loughborough, LE11 3TU, UK; G.Vinner@lboro.ac.uk

**Keywords:** bacteriophage, *Clostridium difficile*, phylogenetic analysis, CRISPR/Cas system, genome evolution, endolysin, large terminase gene

## Abstract

Bacteriophages (phages) are increasingly being explored as therapeutic agents to combat bacterial diseases, including *Clostridium difficile* infections. Therapeutic phages need to be able to efficiently target and kill a wide range of clinically relevant strains. While many phage groups have yet to be investigated in detail, those with new and useful properties can potentially be identified when phages from newly studied geographies are characterised. Here, we report the isolation of *C. difficile* phages from soil samples from the north of Iraq. Two myoviruses, CDKM15 and CDKM9, were selected for detailed sequence analysis on the basis of their broad and potentially useful host range. CDKM9 infects 25/80 strains from 12/20 *C. difficile* ribotypes, and CDKM15 infects 20/80 strains from 9/20 ribotypes. Both phages can infect the clinically relevant ribotypes R027 and R001. Phylogenetic analysis based on whole genome sequencing revealed that the phages are genetically distinct from each other but closely related to other long-tailed myoviruses. A comparative genomic analysis revealed key differences in the genes predicted to encode for proteins involved in bacterial infection. Notably, CDKM15 carries a clustered regularly interspaced short palindromic repeat (CRISPR) array with spacers that are homologous to sequences in the CDKM9 genome and of phages from diverse localities. The findings presented suggest a possible shared evolutionary past for these phages and provides evidence of their widespread dispersal.

## 1. Introduction

*Clostridium difficile* is a Gram-positive, spore-forming, anaerobic bacterium that can cause infection (termed *C. difficile* infection: CDI) and, in severe cases, lead to pseudomembranous colitis [1,2]. CDI usually occurs as a result of antibiotic treatment that decreases the diversity of the intestinal microbiota, allowing *C. difficile* to proliferate and cause infection [3]. In the last 15 years the epidemiology of CDI has changed considerably due to the rapid emergence of hypervirulent strains, which has raised concerns over its evolution, pathogenicity and antibiotic resistance [4,5].

The natural resistance of *C. difficile* to multiple antibiotics has prompted researchers to investigate phage therapy to combat CDI [6,7,8]. Several *C. difficile* phages from both the *Myoviridae* and *Siphoviridae* families have already been characterised, including by genome sequencing [9,10,11,12,13,14,15,16,17]. These phages are currently classified into two genera, phicd119virus [18] and the proposed phiMMP04virus [19] (both in the *Myoviridae* family), however there are other *C. difficile* phages that do not fall into either genera [13,16,17]. Previous attempts to classify *C. difficile* myoviruses suggested that they can be grouped according to their particle morphology (by tail length and capsid diameter). This groups the long tailed myoviruses, medium myoviruses and small myoviruses, where each group has a shared gene content and genome architecture [11,19].

The aim of this work was to expand our knowledge of existing *C. difficile* phage diversity by isolating and characterising phages from the little studied soil and sediment ecosystems of the north of Iraq. Two myoviruses, CDKM9 and CDKM15, were isolated and characterised according to their host range and genome sequences. They both infect several clinically relevant strains, and were selected for further investigation as candidate therapeutic phages from a larger set of viruses based on their host range. Their genomes were analysed with known *C. difficile* phages to determine their overall relatedness and to inform their potential inclusion within phage cocktails developed for therapeutic use.

## 2. Materials and Methods

### 2.1. Phage Isolation

To isolate phages, soil and sediment samples were taken from sites across mountains and river banks in the north of Iraq. The samples were collected in March 2013 from a depth of 10–20 cm, and stored at 4 °C. The method for phage isolation was followed as described previously [20]. Briefly, the samples were suspended in 10 mL of fastidious anaerobic broth (FA: Bioconnections, Leeds, UK) supplemented with 250 µg·mL^−1^ cycloserine and 8 µg·mL^−1^ cefoxitin (as selective agents) (Bioconnections, Leeds, UK) and 0.1% sodium taurocholate (Sigma-Aldrich, Dorset, UK) for spore germination. These enrichment cultures were incubated for 10 days in a MiniMACS anaerobic chamber (Don Whitley Scientific, West Yorkshire, UK) at 37 °C under anaerobic conditions (10% H_2_, 10% CO_2_ and 80% N_2_). Following incubation, the cultures were centrifuged for 10 min at 3398 × *g*. The supernatants were filtered through 0.22 μm filters and the phages were isolated using 15 *C. difficile* indicator strains (Table S1). Phages were purifed using standard plaque assays after resuspension in Oxoid Brain Heart Infusion broth (BHI; Oxoid Ltd, Basingstoke, UK) and stored in SM buffer (0.1 M NaCl, 1 mM MgSO_4_, 0.2 M Tris-HCl, pH 7.5) [21] 50% glycerol (*v*/*v*) at −80 °C.

The morphology was determined by transmission electron microscopy (performed by Stefan Hyman and Natalie Allcock, Core Biotechnology Services, University of Leicester, UK). Briefly, samples were placed on individual glow discharged pioloform/carbon coated copper grids (Athene type 3 mm: Agar Scientific Ltd, Stansted, UK). Samples were negatively stained with 0.1% uranyl acetate and examined with a a JEOL JEM-1400 electron microscope (JEOL UK Ltd, Welwyn Garden, UK) with an accelerating voltage of 80 kV. Digital images were captured using an SIS Mega view III Digital camera with associated analysis software (Olympus Soft Imaging Solutions, Muenster, Germany).

### 2.2. Phage Host Range Assay

A standard spot test method was used to determine phage host range using phage stocks of 10^8^ plaque-forming units (PFU) mL^−1^ as follows. 250 μL of overnight culture of each of the bacterial strains was mixed with 3 mL of BHI 0.5% agar supplemented with salt solution (0.4 M MgCl_2_ and 0.01 M CaCl_2_) and then poured onto a BHI 1% agar plate [22]. When the agar solidified, 10 μL undiluted drops of each phage were spotted onto its surface, performed in triplicate. Spots were inspected for lysis (a clearing of the bacteria) after 24 h incubation at 37 °C under anaerobic conditions. The spot tests were performed in triplicate.

### 2.3. Purification of Phage Genomic DNA

The two phages, CDKM15 and CDKM9, were isolated on *C. difficile* strain CD105HE1 (R076, equine isolate) [21], and propagated in a liquid culture to obtain a high titre phage stock (10^9^ PFU mL^−1^). Phage genomic DNA was extracted for sequencing using standard phenol chloroform extraction and isopropanol precipitation methods [23] with modifications as follows. The crude lysate of 10^9^ PFU mL^−1^ was centrifuged and filtered through a 0.22 μm filter, treated with 1.4 µg·μL^−1^ DNase, 3 µg·µL^−1^ RNase (Sigma-AldrichTh) and 12.5 μL 1 M MgCl_2_ (Acros Organics, Morris Plains, NJ, USA), and incubated overnight at 37 °C. Proteinase K (Fisher Scientific UK Ltd, Loughborough, UK), ethylenediaminetetraacetic acid (EDTA) (Sigma-Aldrich, Dorset, UK) and sodium dodecyl sulfate (SDS) (Sigma-Aldrich) were added to a final concentration of 0.5 mg·mL^−1^, 20 mM and 0.5%, respectively. This was incubated at 55 °C for one hour. To obtain purified DNA, three rounds of phenol:chloroform:isoamyl alcohol (25:24:1) purifications were performed. The resulting fraction was treated with 0.3 M sodium acetate (Fisher Scientific UK Ltd) and two volumes of ice-cold 95% ethanol to precipitate the DNA, followed by a 10 min incubation on ice. The DNA was pelleted by centrifugation at 21,000× *g* for 20 min and the pellet was washed once with 0.5 mL of 70% ethanol before resuspension in an elution buffer (5 mM TrisCl, pH 8.5). DNA quantity and quality were measured using a Nanodrop 2000 and Qubit Fluorimeter (Thermo Scientific, Loughborough, UK) as described here [6].

### 2.4. Phage Genome Sequencing

The genome of CDKM15 was sequenced at Beijing Genomics Institute (BGI; Shenzhen, China). A paired-end library was prepared using 3 μg of DNA with an insert size of 170 bp and sequenced using an Illumina HiSeq 2000 (San Diego, CA, USA).

CDKM9 was sequenced at Warwick University, UK. An amount of 1 ng of input DNA was used to prepare a paired-end library using an Illumina Nextera XT DNA sample kit according to the manufacturer’s protocol. Sequencing was performed on an Illumina MiSeq using the paired-end 2 × 250 bp protocol (version 2, 500 cycles).

Sequencing read quality was checked with FastQC version 0.11.3 [24] and reads were trimmed using sickle [25]. The reads were assembled using SOAP denovo 2.04 [26,27], SPades 3.1 [28] and Geneious 9.0.5 [29]. Phage sequences assembled into a single contig/gapless scaffold each time, except one case using SOAP which generated two contigs for CDKM9. Independent assemblies were compared and their quality was assessed by mapping reads back to each contig in Geneious using its read mapping algorithm (using “medium” and “medium–low” setting packages). Uncertain or ambiguous regions were resolved by manual inspection of the read mapping and, if needed, by PCR amplification and Sanger sequencing (carried out at GATC Biotech Ltd., London, UK). For tool settings and assembly statistics, see Table S2.

Protein coding genes were predicted using GeneMarkS, GeneMark.hmm [30], Glimmer 3 [31], RAST [32], FGENESV (Softberry, Inc., Mount Kisco, NY, USA) and Prodigal 1.20 [33]. Coding DNA sequences (CDSs) with no overlapping BLASTx hits (against the National Center for Biotechnology Information (NCBI) non-redundant (nr) database) and predicted by only a single tool were discarded. Conflicting start codons were resolved based on BLAST alignment and ribosome binding site (RBS) positions (located by scanning the whole genome with find individual motif occurrences (FIMO) tool using consensus RBS motif found in 5′ untranslated regions (UTRs) of uncontested genes with Multiple Em for Motif Elucidation (MEME) [34] (see Table S2). Results of the BLASTx analyses were also used for the functional annotation of CDSs (we manually assessed the top 50 hits against nr and RefSeq databases to find the most probable function of each protein). Predicted CDS were translated and their initial annotation was re-assessed using BLASTp, InterProScan 5 and CD-Search [35,36,37]. tRNA genes were predicted by tRNAscan-SE version 1.21 and other non-coding RNAs by Infernal 1.1.1 [38,39]. Clustered regularly interspaced short palindromic repeats (CRISPR) arrays located by Infernal were confirmed by PILER-CR 1.06 and CRISPRFinder [40,41]. CRISPRTarget was used to identify matches to the spacers in the array (match reward +1, mismatch penalty −5, minimum score 25) and identify protospacer adjacent motifs (PAMs) [42].

Each genome was oriented to start at the terminase small subunit gene to be consistent with previously sequenced *C. difficile* phage genomes [9,10,12] and deposited in GenBank under the accession numbers KX228399 (CDKM9) and KX228400 (CDKM15).

### 2.5. Phylogenetic Analyses and Comparative Genomics

To determine the taxonomic relatedness of CDKM15 and CDKM9, genome comparisons were performed using Gegenees 2.2.1 [43] to other phages infecting *C. difficile* (reference phage genomes used are listed in Table S3). Gegenees calculates global similarity between pairs of sequences based on BLAST local alignments (we used both the BLASTn and BLASTx method with a fragment size of 200, sliding window size of 100). The resulting BLASTx similarity matrix was used to construct BioNJ phylograms with SplitsTree 4.13.1 [44].

A phylogenetic tree was generated using maximum likelihood (ML) analysis of the endolysin genes at the amino acid level to determine whether phage genes involved in host cell lysis share the same evolutionary history of the phage genomes overall. Homologous endolysin sequences were retrieved from GenBank using BLASTp and scanned for relevant domains using InterProScan in Geneious (Table S3) [29]. Verified sequences were aligned using ClustalW in Geneious, evolution models were selected with ProtTest 3.2.1 and ML analysis performed using PhyML 3.0 [45].

To predict the packaging strategy of analysed phages, we followed the method proposed by Casjens and Gilcrease [46]. The terminase large subunit genes from 24 *C. difficile* phages and 68 reference phages (Table S3) were aligned at the amino acid level using ClustalW in Geneious. FastTree 2.1.7 was used to generate approximate ML tree, run with the Whelan and Goldman (WAG) substitution model (selected using of ProtTest 3.4) and the computation of gamma likelihoods enabled and the Shimodaira–Hasegawa test used to calculate support values for the nodes [47]. This method is much faster than classic ML with only a negligible loss of topological accuracy [48]. Trees were visualised in Geneious.

Genome comparisons were performed using Blast Ring Image Generator (BRIG) v.0.95 [49] and EasyFig 2.2.2 [50] which displays results of BLASTn-based sequence comparisons based on pairwise similarity of the matches in circular and linear representations, respectively.

### 2.6. Protein Analysis

Protein cluster analysis was performed to determine the fraction of shared proteins between CDKM9, CDKM15 and 22 *C. difficile* phages. Protein clusters were created using CD-HIT [51] and protein cluster statistics were generated in Microsoft Excel.

## 3. Results

### 3.1. Phage Isolates and Host Range Analysis

**Figure 1:**
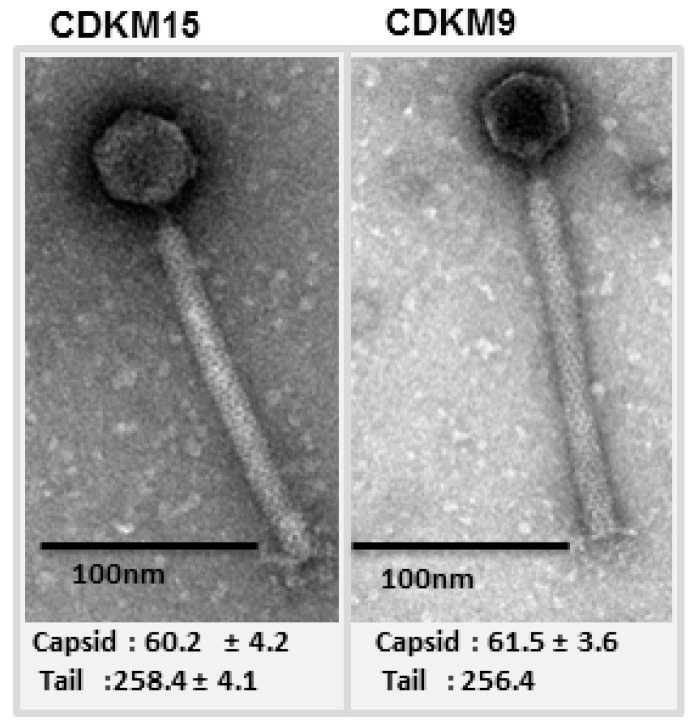
Transmission electron micrographs of phages CDKM15 and CDKM9. The sizes of the capsids and tails were measured for four particles and the mean values in nm with the standard deviations are reported below each phage.

**Figure 2:**
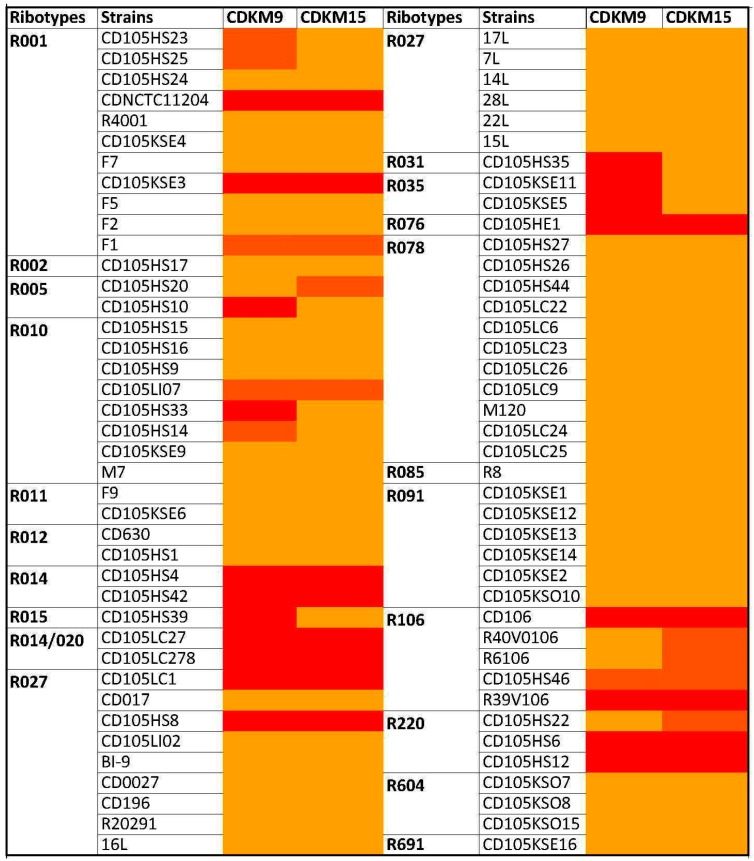
Heat map representation of lysis profiles of CDKM9 and CDKM15 for 80 *Clostridium difficile* strains. Colours indicate different phage infection parameters observed in the spot tests: red is clearing of the lawn, orange is clearing with turbidity and yellow was no clearing.

To explore the biology of *C. difficile* phages in the north of Iraq, soil and sediment samples were collected from different sites which were then used for phage isolation using 15 indicator strains from four PCR ribotypes: R027, R078, R010 and R076 (Table S1). Fourteen phages were isolated and belonged to either the *Siphoviridae* (12/14) or *Myoviridae* (2/14) based on particle morphology as observed using transmission electron microscopy (TEM). All the *Myoviridae* displayed the characteristically long tails of the previously categorised long tailed myoviruses [29] (see Figure 1 for example). The phage host ranges were tested on 80 *C. difficile* isolates (Figure 2, Table S4). The panel of strains represented 20 ribotypes and originated from four countries: Kurdistan in the north of Iraq (*n* = 22), the UK (*n* = 55), France (*n* = 1), Switzerland (*n* = 1) and USA (*n* = 1). The strain panel included clinical isolates (*n* = 31), environmental isolates (*n* = 46), asymptomatic infant isolates (*n* = 2) and a single bovine isolate. Two myoviruses, CDKM15 and CDKM9 (Figure 1), were selected for further characterisation as they had the broadest host ranges from the new set of phages and, in particular, could lyse *C. difficile* isolates from the ribotype R027.

The host range analysis showed that CDMK15 infected 20/80 strains (25% tested) from 9/20 ribotypes, and CDMK9 infected 25/80 strains (31% tested) from 12/20 ribotypes. Also, a broad phage host range overlap was evident, as 16 isolates were infected by both CDKM9 and CKM15, which were represented in nine ribotypes. Notably, isolates belonging to R015, R031 and R035 were infected exclusively by CDKM9 (Figure 2). Overall, both phages infected strains from different environments and different sources. Furthermore, both phages infected strains isolated from Kurdistan, the ‘local isolates’. Of particular interest, due to their potential clinical utility, the phage CDKM9 infected four strains from R001 and R010, and CDKM15 infected two strains of R001 (Figure 2, Table S4).

### 3.2. Genome Features of CDKM15 and CDKM9

**Figure 3:**
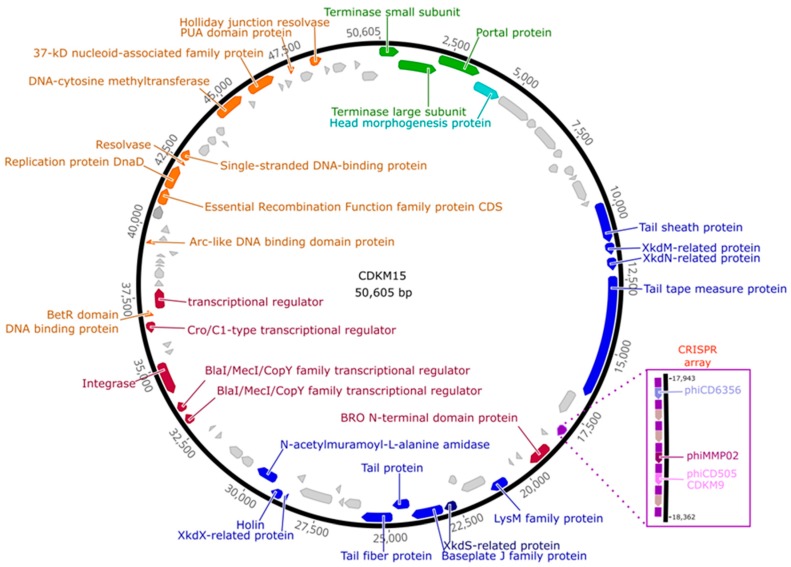
Genome organisation of CDKM15. The 50,605 bp genome assembled as a circle, shown here oriented to start at the terminase small subunit gene. Predicted coding DNA sequences (CDSs) are marked with arrows and colours indicate functional modules: head packaging (green), head (aquamarine), tail and lysis (blue), lysogenic conversion (purple) and DNA replication (orange). CDSs with no function assigned are light grey. Functional annotations are labelled. The clustered regularly interspaced short palindromic repeats (CRISPR) array is marked with a mauve arrow. The CRISPR array and its spacers are highlighted with spacer matches indicated.

The dsDNA genomes of CDKM15 and CDKM9 are 50,606 bp and 49,822 bp, respectively. Both phages have a GC content of 28.98%, which is similar to that of published *C. difficile* phages, and to the reference *C. difficile* strain CD630 (29.06%) [52]. In total, the genome of CDKM15 had 79 predicted CDSs, with 73 on the sense strand and six on the antisense. Of these 79 CDSs, 34 (43%) had a predicted function assigned (with a BLASTp e-value of 1 × 10^−10^ as a cut-off) and 45 (57%) encode for genes with an unknown function (Figure 3 and Table S2). 

**Figure 4:**
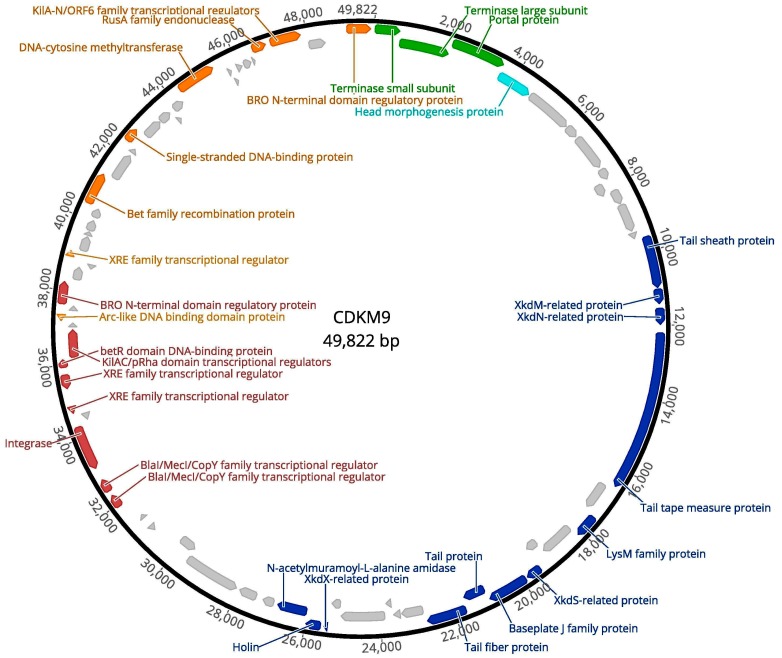
Genome organisation of CDKM9. The 49,822 bp genome assembled as a circle, shown here oriented to start at the terminase small subunit gene. The predicted CDSs are marked with arrows and colours indicate functional modules: head packaging (green), head (aquamarine), tail and lysis (blue), lysogenic conversion (purple) and DNA replication (orange). CDSs with no function assigned are light grey. Those with functional annotations are labelled.

The genome of CDKM9 had 75 predicted CDSs, with 66 on the sense strand and nine on the antisense strand. Of the 75, 32 (42.7%) could be assigned putative functions, and 43 (57.3%) could not (Figure 4, Table S2).

Following the genome annotations, it was apparent that the phage genomes displayed a clear modular organisation. There are distinguishable gene modules whose products are predicted to be involved in DNA packaging, virion assembly, host cell lysis, lysogeny control and DNA replication. No tRNA genes were identified in either genome. Each genome had a lysogeny control gene module with a predicted integrase, and two copies of *repR*, a predicted regulatory protein containing a penicillinase repressor family protein domain (Pfam: PF03965).

Interestingly, CDMK15 has a CRISPR array located adjacent to a cluster of genes with unknown function preceding the tail morphogenesis module, including a CDS with a predicted baculovirus repeated open reading frame (Bro) N-terminal domain protein (Pfam: 02498) (Figure 3). The array contains six 34–37 bp long spacers and seven 29 bp direct repeats (DRs). There are no *cas* genes in the genome, a finding which is consistent with previously analyses of CRISPR arrays in *C. difficile* phages [11]. The first five DRs (from the 5′ end of the array) are identical, while the last two harbour two and seven mutations (compared to the consensus sequence), respectively. The array is preceded by a 215 bp leader region that shares similarity to that of CRISPR array no. 14 in *C. difficile* R20291 (71% similarity in pairwise ClustalW alignment, 100% in conserved 13-bp 3′ motif) [53,54]. The consensus DR sequence (ATTTTATATTAACTATGTGGTATGTAAAT) differs by four nucleotides to that of the DRs in prophage 1 from CD630 (GTTTTAGATTAACTATATGGAATGTAAAT). All spacer sequences are unique to CDKM15 when searched against *C. difficile* isolate sequences in NCBI. Of the six spacers, spacer 1 was found to perfectly match to the genome sequence of ΦCD6356, spacer 5 imperfectly matched to ΦCD505 and CDKM9 (97% identity), and spacer 4 imperfectly matched to ΦMMP02 (94.6% identity). A protospacer adjacent motif (PAM) of CCN (A or T) has been predicted for *C. difficile* [11,53]. We observed CCN PAMs in the phage genomes for all matches with the exception of the non-identical match to ΦMMP02. The PAM sequence is required for functional targeting, and our findings support the hypothesis that the phage carried spacers may be able to provide functional immunity against the corresponding phages.

### 3.3. Phylogenetic Analyses

**Figure 5:**
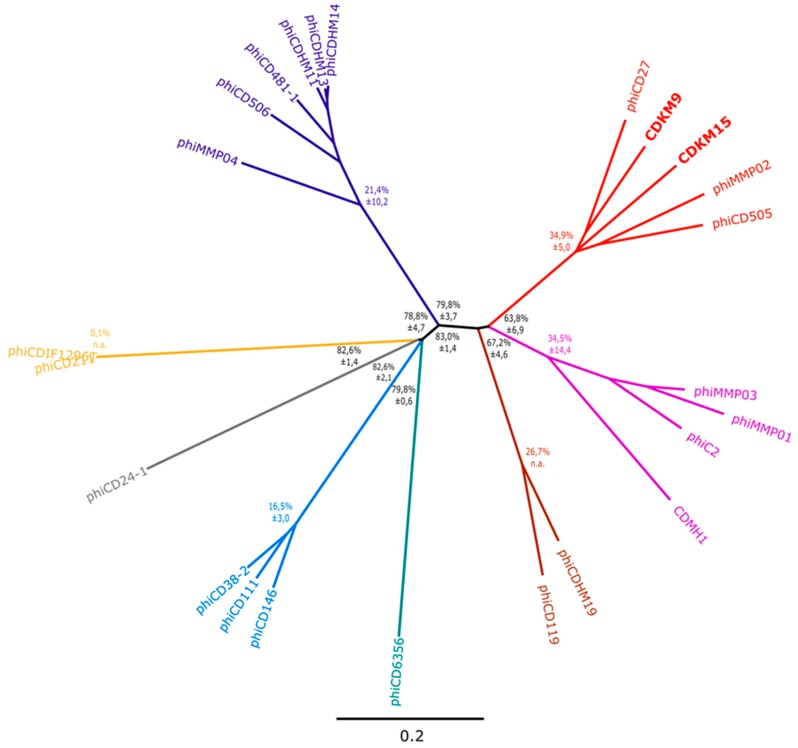
Phylogenetic tree based on whole genome comparison of *C. difficile* phage genomes. The similarity values were calculated based on a translated pairwise comparison of the analysed sequences using Gegenees software. The phylogenetic tree was constructed with SplitsTree using the neighbor joining method. The scale bar represents a 20% difference in average tBLASTx score. Branch colours correspond to the colours in Figure 6. Black node markers represent mean percentage distance between the clades (calculated by averaging each distance between group 1 member and group 2 member) ± standard deviation. Coloured node markers represent the mean percentage distance within the clade (calculated by averaging distances between group members) ± standard deviation. Distance is defined as 100% similarity in translated whole genome comparison.

In order to determine how the newly isolated phages are related to previously described *C. difficile* phages, we constructed a phylogenomic tree based on values of nucleotide sequence pairwise similarity between CDKM9, CDKM15 and 22 *C. difficile* phages. The resulting tree revealed that CDKM9 and CDKM15 group with the long tailed myoviruses ΦCD27, ΦCD505 and ΦMMP02 (Figure 5). There may be a distant evolutionary relationship between the two clusters of medium myoviruses (one of them corresponding to the currently accepted genus phicd119virus), but overall similarity between genomes of these groups fell below 40% on the nucleotide level and 50% for translated comparison (Table S5). The “jumbo” myoviruses (ΦCD211 and ΦCDIF1296T) were even more divergent and could not be classified into any of these groups. As might be expected, siphoviruses clustered together, but sub-clusters were observed for the phages that split into a group containing ΦCD38-2, ΦCD111 and ΦCD146, and two singletons ΦCD24-1 and ΦCD6356.

### 3.4. Phylogeny of the *Endolysin* Genes

**Figure 6:**
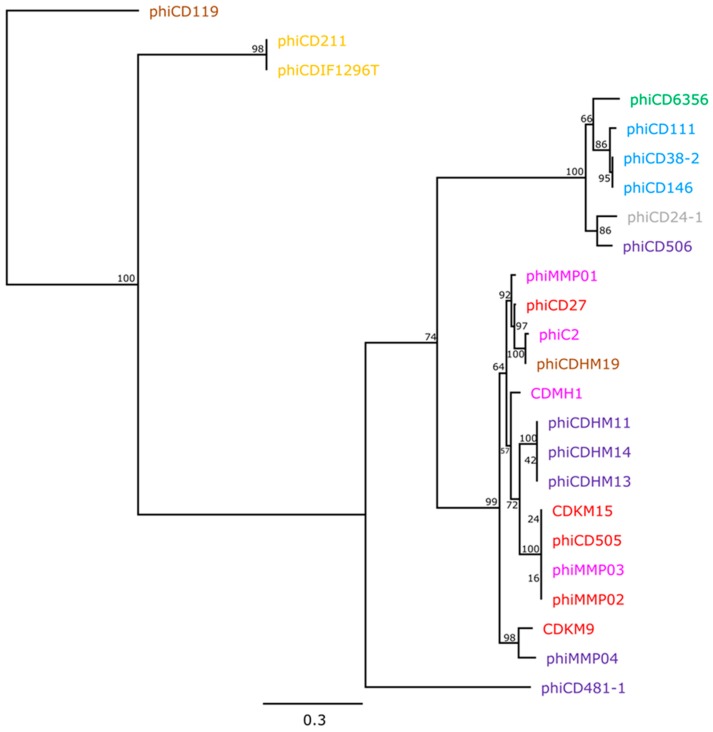
Maximum likelihood phylogenetic analysis of *C. difficile* phage endolysins. Tree node labels represent bootstrap values.

Phage endolysins are required for lysis of the cell wall, and all sequenced *C. difficile* phages encode an endolysin containing *N*-acetylmuramoyl-l-alanine amidase domains. Phylogenetic analysis was performed on endolysin sequences to establish whether this gene is subject to horizontal gene transfer (HGT), or follows the same evolutionary trajectory as the phage genome. To do this, ML analysis was applied to endolysin sequences from 24 phages (Figure 6). The resulting tree showed that taxa grouped into clades largely reflect the taxonomic division between siphoviruses and myoviruses, in which 15 taxa are myoviruses, and six are siphoviruses clade. However, the topology of the endolysin tree is incongruent with that of the whole genome based tree, as the myoviruses were clustered in such a way that did not reflect their particle morphology (unlike the case for the whole genome phylogeny). Evidence of HGT of the endolysin can be seen for ΦCD506 which clustered with the siphoviruses highlighting this mechanism as facilitating phage genome evolution. 

### 3.5. Phylogenetic Analysis of *terL* and the Packaging Strategy of the Isolated Phages

**Figure 7:**
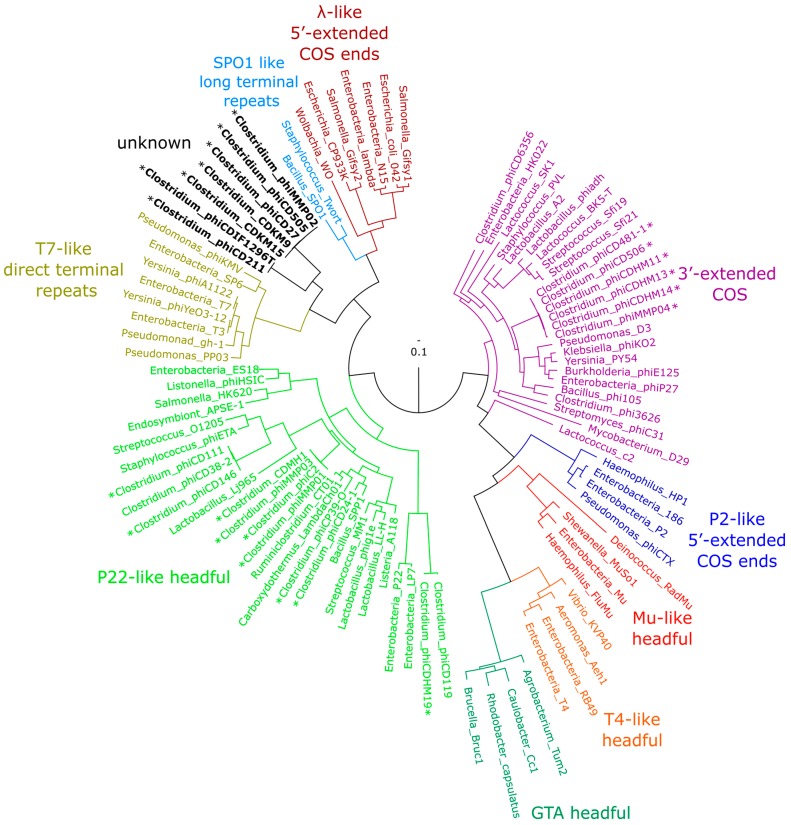
Phylogenetic tree of phage terminase large subunit (*terL*) gene. The name of the phage or prophage is shown at each terminal node and the packaging strategy for each group is labelled where known. The branches are coloured according to the DNA packaging strategy: purple (3′-cohesive ends), blue (5′-cohesive ends), red (Mu-like headful), orange (T4-like headful), jade (GTA headful), green (P22-like headful), olive (T7-like direct terminal repeats), black (unknown), light blue (SPO1 long terminal repeat) and maroon (λ-like 5′-extended COS ends). An asterisk (*) next to a taxon label indicates phages with a packaging strategy predicted during this analysis or earlier predictions using similar methodology, but without experimental evidence (to the best of our knowledge).

To determine the packaging mechanism for CDKM15 and CDKM9, we followed a method described by Casjens and Gilcrease [46]. A phylogenetic tree was generated for *terL* which encodes the terminase large subunit (Figure 7). CDMK9 and CDMK15 form a clade with ΦCD505, ΦCD27 and ΦMMP02 which is consistent with the whole genome tree results. These phages do not cluster with any phages that have a predicted or experimentally confirmed packaging strategy. In an attempt to identify a predicted packaging strategy, we performed read mapping to identify distinct genome termini but did not detect a signal indicating the location of these termini (e.g., *cos* sites). While we did not detect these termini it is important to remember that fragments generated during library preparation may not be entirely random and their uneven distribution may hinder end analysis (this may be especially true for the Nextera libraries used to sequence CDKM9).

### 3.6. Comparative Genomics

**Figure 8:**
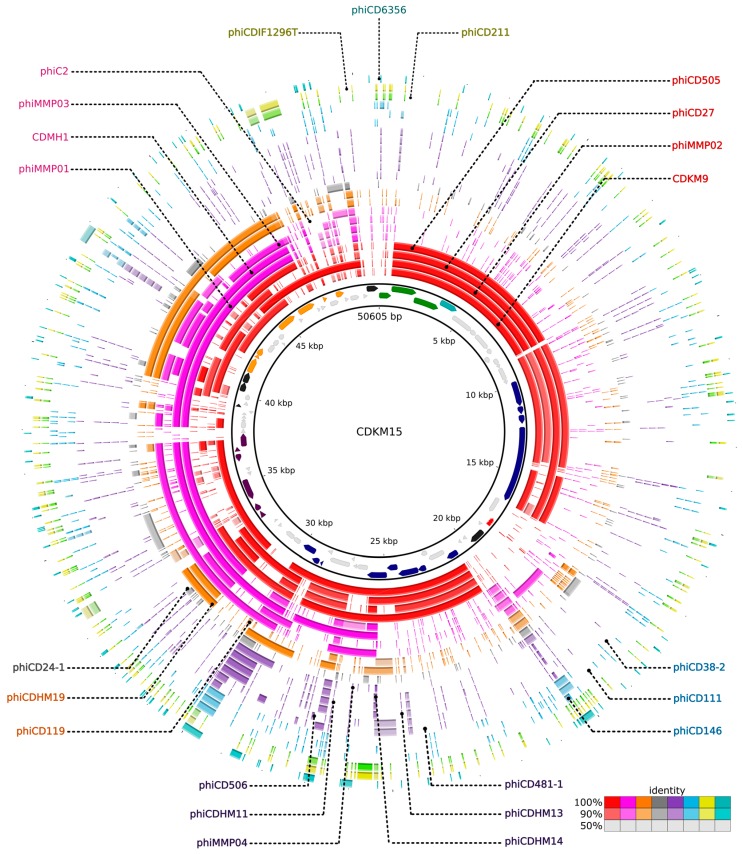
Whole genome comparison of CDKM15 and *C. difficile* phages. The local similarity of each phage is calculated based on BLASTn high scoring pairs and plotted against a circular map of the reference genome represented as the inner circle (in this case genome of CDKM15). Similarity to each of the 23 *C. difficile* phages is shown as colouring intensity in consecutive rings. Outer rings are coloured consistent to the scheme in Figure 6.

CDKM9 and CDKM15 were each used as a reference to which 22 phage sequences were compared by BLASTn in order to visualise regions sharing similarity across the *C. difficile* phage genomes (Figure 8, Figure S1 and S2). In the resulting maps, the three long-tailed myoviruses (LTM) (ΦCD505, ΦCD27 and ΦMMP02) display the most similarity to CDMK9 and CDKM15. Regions of similarity were observed in the packaging and structural modules, but to a lesser extent in the lysogeny control and DNA replication modules. Instead, here, the viruses are more similar to the medium myoviruses (ΦC2, ΦCDHM1, ΦMMP03, ΦMMP01, ΦCDHM19 and ΦCD119). As might be expected, the genetic variability of the modules varied to different degrees; the lysogeny control region is divergent across the genomes, while the cluster of genes responsible for lysis is conserved across the 24 phages, which can be expected considering they all infect the same host species.

### 3.7. Protein Cluster Analysis

**Figure 9:**
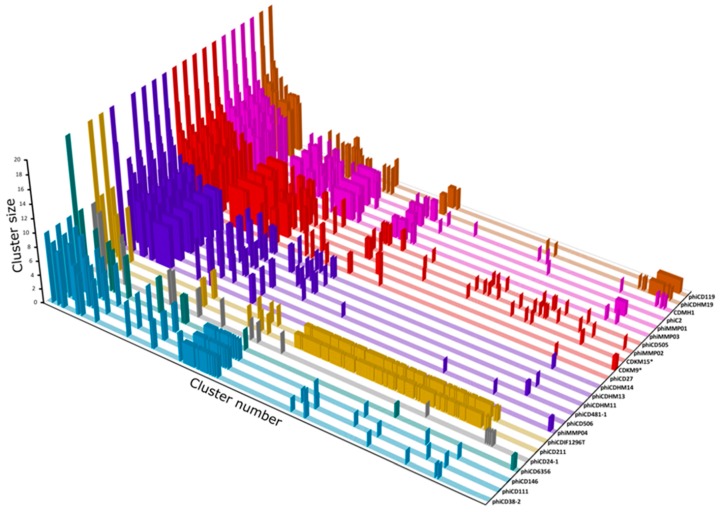
Plot of protein clusters shared by the 24 *C. difficile* phages. The *y*-axis represents the cluster size (number of phages encoding homologues) and clusters are arranged along the *x*-axis by size. Colouring is consistent with Figure 6.

Cluster analysis was performed on 1850 predicted proteins from 24 phage genomes to identify the core genes shared by all *C. difficile* phages and those which are specific to groups or particular phages. The protein sequences were grouped into 479 clusters, with 229 singletons (Figure 9, Table S5). Interestingly, the most prevalent cluster (cluster no. 0 with sequences from 19 phages) was comprised entirely of protein sequences with no known function. The gene was conserved amongst 18/19 myoviruses and a single siphovirus, ΦCD6356 and usually located between putative tail fiber and endolysin genes. Thus, we hypothesise that the CDS product may be involved in phage-host attachment or interaction. Almost all endolysin sequences grouped into two clusters that corresponded to a family level division for the phages (14/19 myoviruses had endolysins from cluster no. 1, while all five siphovirus endolysins grouped to cluster no. 47). Exceptions to this were the endolysin of myovirus ΦCD506 which clustered with the siphoviruses, (which is consistent with results of the phylogenetic analysis) and the endolysins of “jumbo” myoviruses (ΦCDIF1296T and ΦCD211) which formed a separate cluster.

Generally, the protein clusters were confined to phage family (siphovirus or myovirus) or a subgroup, but CDSs in 41/708 clusters were not. These include the above-mentioned cluster no. 0 and a further five clusters that contain CDSs from the siphoviruses and the short tailed myoviruses (clusters no. 5, 7, 17, 21and 25 which are carried in the replication modules of at least eight phages). 

Lastly, this analysis revealed four proteins unique to CDKM9, and 10 to CDKM15. For CDMK9, these are three proteins of unknown function (CDS 59, 67, and 68) and a predicted recombination protein with a lambda Bet-like RecT superfamily domain [55,56] encoded in the DNA replication region. For CDKM15, the unique CDS encodes eight hypothetical proteins (CDS 11, 18, 19, 21, 22, 50, 78, and 79), a predicted anti-repressor (CDS 49) and its terminase small subunit (CDS 1).

## 4. Discussion

Before this work all phages that infect *C. difficile* were isolated from sources within Europe and North America. In this study, we extend the global picture of their diversity by isolating phages from northern Iraq. In total, 14 phages were isolated and two phages further characterised. CDKM9 and CDKM15 could infect 18% (4/22) (CDKM9) and 9% (2/22) (CDKM15) ‘local’ isolates from Kurdistan (north of Iraq), and 36% (21/58) (CDKM9) and 31% (18/58) (CDKM15) of ‘global’ isolates from the UK. Their host ranges include isolates from ribotypes associated with major epidemics: R027, R001, R014, R014/020 and R005 [57,58,59], however, no isolates of R078 were sensitive to either phage. An overlap in each phage’s host range was observed, with 16 of 25 and 20 infected by CDKM9 and CDKM15, respectively, suggesting they likely use the same receptor [22]. Both the genome organisation and particle morphology of CDKM9 and CDKM15 resemble that of the long tailed myoviruses ΦCD27, ΦCD505 and ΦMMP02. Interestingly, however, CDKM9 has the broadest reported host spectrum compared to the reported host ranges of other long tail myoviruses (ΦCD27, ΦCD505, ΦCD508, ΦCDHM2, ΦCDHM4, ΦCDHM5 and ΦCDHM6) as these infected 13% (4/30), 11% (5/47), 4/47, (28%) 22/80, 4/80 (5%), 20/80 (25%), (29%) 23/80 isolates, respectively [6,14,16]. Further, no other long tailed myoviruses have been shown to lyse R027 isolate [6,14,16,60], while siphoviruses infecting R027 strains have been identified [6,16,17] and the only other report in the literature of myoviruses with this ability are the medium myoviruses ΦCD481-1 and ΦCDHM3, both of which caused turbid plaques on a single strain of R027 each [6,16].

The genomes of CDKM9 and CDKM15 were sequenced to determine their taxonomic relationships to other *C. difficile* phages. While the genome lengths and GC contents are similar to those values specified for inclusion into the species phicd119virus [18], at just ~50 kb their genomes are at the lower end of the 51–60 kbp range. To further characterise these phages, their shared protein content was analysed (see below).

Annotation of the two phage genomes revealed a notable feature of phage CDKM15; it encoded a CRISPR array. *C. difficile* strains carry multiple CRISPR arrays including those which, based on the *cas* gene content, belong to type I-B/Tneap classification [11,52,61]. In addition to arrays on the main chromosome, arrays have been identified on mobile genetic elements including the skin^cd^, prophages and a plasmid [11]. The prophage carried arrays do not have obvious *cas* genes but do encode proteins predicted to have DNA binding ability [11]. Here, we identified arrays on a phage that had been propagated via lytic replication on its indicator host strain. The two prophages found in CD630 both contain CRISPR arrays and can be propagated through lytic infections [9], however whether the CRISPR arrays were maintained during these cycles was unknown, but CRISPR loci had been discovered on a related phage from free viruses in the human gut [62] and in the induced viral particle of prophage in CD105HSE1 [11]. Consistent with the previously characterised phage arrays, the array in CDKM15 is located in the region involved in tail morphogenesis near the gene encoding Bro N-terminal protein [11]. The structure of the array is similar to those found to be active and expressed arrays in *C. difficile* in terms of crucial leader motifs, direct repeat sequences and spacer lengths [11,53] but it has unique spacer content. As CDKM15 was isolated from a poorly studied environment, we wanted to determine if its spacer sequences might be derived from phages isolated in the UK and USA. Indeed, one spacer perfectly matches to ΦCD6356, and other spacers imperfectly match to sequences from phages ΦCD505, CDKM9 and ΦMMP02 which could suggest that there is a global dissemination of strains and phages. The finding of a CRISPR array in CDKM15 expands our understanding of how phages might transfer CRISPR arrays (and resulting immunity) to sensitive cells. Furthermore, the finding that spacers match to other phage genomes, including the ‘neighbouring’ (CDMK9) and globally distributed phages, such as ΦCD6356 isolated in the Ireland, are evidence of past co-infection events which resulted in acquisition of these spacers. These findings suggest that either long standing evolution and/or subsequent dispersal of similar phages have occurred at a global scale within *C. difficile*.

The results of the comparative genomic analysis of the 24 *C. difficile* phages highlight the mosaic nature of their genomes (Figure 8, Figure S1 and S2). All phage genomes contained a predicted endolysin gene and its phylogenetic analysis suggested that it has undergone HGT, exemplified by the *endolysin* gene of the myovirus ΦCD506 as it is in a clade with siphovirus *endolysin* genes. It seems likely that exchange of this gene can occur between the different phages and host genomes during co-infection, but stabilising selection, driven by the need to recognise the host wall, prevents protein sequences from diverging [63]. Polylysogeny in *C. difficile* is known, for example the sequenced genome of CD630 contains two related prophages, CD630-1 and CD630-2 [52] and is evident from PCR based screens and the observations of multiple different phage particle morphologies in culture lysates [16,21,64,65,66]. Co-infection with multiple phage types clearly presents the opportunity for gene exchange. 

Phage *terL* sequences have been used previously to reconstruct phylogenic relationships [67]. Moreover, Casjens and Gilcrease [46] demonstrated that this sequence may be used to predict the phage’s DNA packaging strategy. One of the considerations of using phages for therapeutic purposes is their ability to facilitate HGT via transduction [68]. The mechanism of DNA packaging into the virion determines how this might occur [69]. Headful packaging (*pac*) phages may perform generalised transduction, but phages with cohesive end (*cos* phages) require sequence recognition in the packaging process. In *C. difficile* phages, ΦCD38-2 has been found to contain a *pac* site, whereas ΦCD6356 has a *cos* site identified [13,17]. However, attempts failed to identify cohesive ends for both ΦCD119 and ΦCD27 [10,14], and no mechanism has been specified for ΦC2, although this phage is capable of performing transduction [70]. Phylogenetical analysis of *terL* showed that the genes from CDKM9 and CDKM15 clustered with phages whose packaging mechanism is unclassified. A second method to predict the DNA packaging mechanism is to identify the termini of the DNA molecule ends, as the *cos* and *pac* strategies result in different sequences [46]. No apparent termini could be located in either CDMK9 or CDMK15, a result which is consistent with the fact that no *cos* sites could be identified in the related phage ΦCD27 [14]. The mechanism of DNA packaging used by this group of related phages therefore remains unknown.

To conclude, phages represent a source of novel antimicrobials and the beneficial properties of ‘therapeutic phages’ include those that are efficient in lysing a wide range of bacterial isolates within the target species. *C. difficile* phages have been investigated from very few countries, and here we demonstrate that phages can be isolated from new sources. Host range analysis of two of these phages suggested they may be of use in the development of phage-based therapeutics. Genome sequencing and analysis revealed new insights into *C. difficile* phage phylogeny and identified signals of HGT. Furthermore, in this work we suggest that the taxonomic framework for classifying this phage group needs to be widened. With this in mind, we propose two genera in addition to phicd119virus, phiMMP04virus, “phiCD38-2virus” containing ΦCD38-2, ΦCD111 and ΦCD146, and “phiCD211virus’’ containing ΦCD211 and ΦCDIF1296T.

## Supplementary Materials

The following are available online at www.mdpi.com/1999-4915/8/11/310/s1, Figure S1: Whole genome comparison of phage CDKM9 and other *Clostridium difficile* phages, Figure S2: Whole genome comparison of all *C. difficile* phages, Table S1: Bacterial strains used in this study, Table S2: Detailed results of the genome assembly, Table S3: Genes of novel *C. difficile* phages and their protein products, Table S4: Sequences and database records used in this study, Table S5: Host range analysis of the examined phages, Table S6: Results of protein cluster analysis and genome comparision.
