# Supplementary material for: Two Novel Myoviruses from the North of Iraq Reveal Insights into Clostridium difficile Phage Diversity and Biology
Source: Viruses. 2016 Nov 16;8(11):310. doi: 10.3390/v8110310 (PMC5127024; doi:10.3390/v8110310)
Supplement: Supplementary file 1 [file viruses-08-00310-s001.zip › viruses-154767-supplementary/Supplemetary files/Supplementary Table S1. C. difficile indicator strain.docx]

**Table S1.** List of *C. difficile* strains used for phage screening.

| **Strains** | **Source** | **Ribotype** |
| --- | --- | --- |
| CD105HS15 | Sediment | 010 |
| CD105HS9 | Sediment | 010 |
| CD105LI07 | Infant | 010 |
| BQT | Human | 010 |
| BQR | Human | 010 |
| K10 | Sediment | 012 |
| CD105LC1 | Human | 027 |
| CD105HS8 | Sediment | 027 |
| CD105LI02 | Infant | 027 |
| CD105LC22 | Human | 078 |
| CD105LC6 | Human | 078 |
| CD105HS44 | Sediment | 078 |
| CD105HS26 | Sediment | 078 |
| CD105HS27 | Sediment | 078 |
| CD105HE1 | Equine | 076 |
