# Supplementary material for: Two Novel Myoviruses from the North of Iraq Reveal Insights into Clostridium difficile Phage Diversity and Biology
Source: Viruses. 2016 Nov 16;8(11):310. doi: 10.3390/v8110310 (PMC5127024; doi:10.3390/v8110310)
Supplement: Supplementary file 1 [file viruses-08-00310-s001.zip › viruses-154767-supplementary/Supplemetary files/Supplementary Table S5. Host range analysis of the examined phages..docx]

Table S4. Host range analysis of the phages examined in this study

| Ribotype | *C. difficile* isolates and origin | | Phages | |
| --- | --- | --- | --- | --- |
|  |  |  | CDKM9 | CDKM15 |
| 001 | CD105HS23 | UK |  |  |
|  | CD105HS25 | UK |  |  |
|  | CD105HS24 | UK |  |  |
|  | CDNCTC11204 | UK |  |  |
|  | R4001 | UK |  |  |
|  | CD105KSE4 | Kurdistan |  |  |
|  | F7 | Kurdistan |  |  |
|  | CD105KSE3 | Kurdistan |  |  |
|  | F5 | Kurdistan |  |  |
|  | F2 | Kurdistan |  |  |
|  | F1 | Kurdistan |  |  |
| 002 | CD105HS17 | UK |  |  |
| 005 | CD105HS20 | UK |  |  |
|  | CD105HS10 | UK |  |  |
| 010 | CD105HS15 | UK |  |  |
|  | CD105HS16 | UK |  |  |
|  | CD105HS9 | UK |  |  |
|  | CD105LI07 | UK |  |  |
|  | CD105HS33 | UK |  |  |
|  | CD105HS14 | UK |  |  |
|  | CD105KSE9 | Kurdistan |  |  |
|  | M7 | Kurdistan |  |  |
| 011 | F9 | Kurdistan |  |  |
|  | CD105KSE6 | Kurdistan |  |  |
| 012 | CD630 | Switzerland |  |  |
|  | CD105HS1 | UK |  |  |
| 014 | CD105HS4 | UK |  |  |
|  | CD105HS42 | UK |  |  |
| 015 | CD105HS39 | UK |  |  |
| 014/020 | CD105LC27 | UK |  |  |
|  | CD105LC278 | UK |  |  |
| 027 | CD105LC1 | UK |  |  |
|  | CD017 | UK |  |  |
|  | CD105HS8 | UK |  |  |
|  | CD105LI02 | UK |  |  |
|  | BI-9 | USA |  |  |
|  | CD0027 | UK |  |  |
|  | CD196 | France |  |  |
|  | R20291 | UK |  |  |
|  | 16L | UK |  |  |
|  | 17L | UK |  |  |
|  | 7L | UK |  |  |
|  | 14L | UK |  |  |
|  | 28L | UK |  |  |
|  | 22L | UK |  |  |
|  | 15L | UK |  |  |
| 031 | CD105HS35 | UK |  |  |
| 035 | CD105KSE11 | Kurdistan |  |  |
|  | CD105KSE5 | Kurdistan |  |  |
| 076 | CD105HE1 | UK |  |  |
| 078 | CD105HS27 | UK |  |  |
|  | CD105HS26 | UK |  |  |
|  | CD105HS44 | UK |  |  |
|  | CD105LC22 | UK |  |  |
|  | CD105LC6 | UK |  |  |
|  | CD105LC23 | UK |  |  |
|  | CD105LC26 | UK |  |  |
|  | CD105LC9 | UK |  |  |
|  | M120 | UK |  |  |
|  | CD105LC24 | UK |  |  |
|  | CD105LC25 | UK |  |  |
| 085 | R8 | UK |  |  |
| 091 | CD105KSE1 | Kurdistan |  |  |
|  | CD105KSE12 | Kurdistan |  |  |
|  | CD105KSE13 | Kurdistan |  |  |
|  | CD105KSE14 | Kurdistan |  |  |
|  | CD105KSE2 | Kurdistan |  |  |
|  | CD105KSO10 | Kurdistan |  |  |
| 106 | CD106 | UK |  |  |
|  | R40V0106 | UK |  |  |
|  | R6106 | UK |  |  |
|  | CD105HS46 | UK |  |  |
|  | R39V106 | UK |  |  |
| 220 | CD105HS22 | UK |  |  |
|  | CD105HS6 | UK |  |  |
|  | CD105HS12 | UK |  |  |
| 604 | CD105KSO7 | Kurdistan |  |  |
|  | CD105KSO8 | Kurdistan |  |  |
|  | CD105KSO15 | Kurdistan |  |  |
| 691 | CD105KSE16 | Kurdistan |  |  |
| Total number of ribotypes = 20 | |  | 12 | 9 |
| Total number of bacterial isolates = 80 | |  | 25 | 20 |

Host range analysis was conducted by applying 10 µl of 10^8^ PFU/ml of phage stocks on lawns of bacteria and incubated anaerobically at 37 ^o^C for 24 h. Key: Black cells= clears, grey cells =turbid clearing, white cells = no clearing. In total, 22 Kurdistan isolates, XX UK isolates, and 1 isolate from each Switzerland, France and USA belonging to 20 ribotypes.
