# Supplementary material for: Two Novel Myoviruses from the North of Iraq Reveal Insights into Clostridium difficile Phage Diversity and Biology
Source: Viruses. 2016 Nov 16;8(11):310. doi: 10.3390/v8110310 (PMC5127024; doi:10.3390/v8110310)
Supplement: Supplementary file 1 [file viruses-08-00310-s001.zip › viruses-154767-supplementary.docx]

Supplementary Materials: Two Novel Myoviruses from the North of Iraq Reveal Insights into
*Clostridium difficile* Phage Diversity and Biology

Srwa J. Rashid, Jakub Barylski, Katherine R. Hargreaves, Andrew A. Millard,
Gurinder K. Vinner and Martha R. J. Clokie


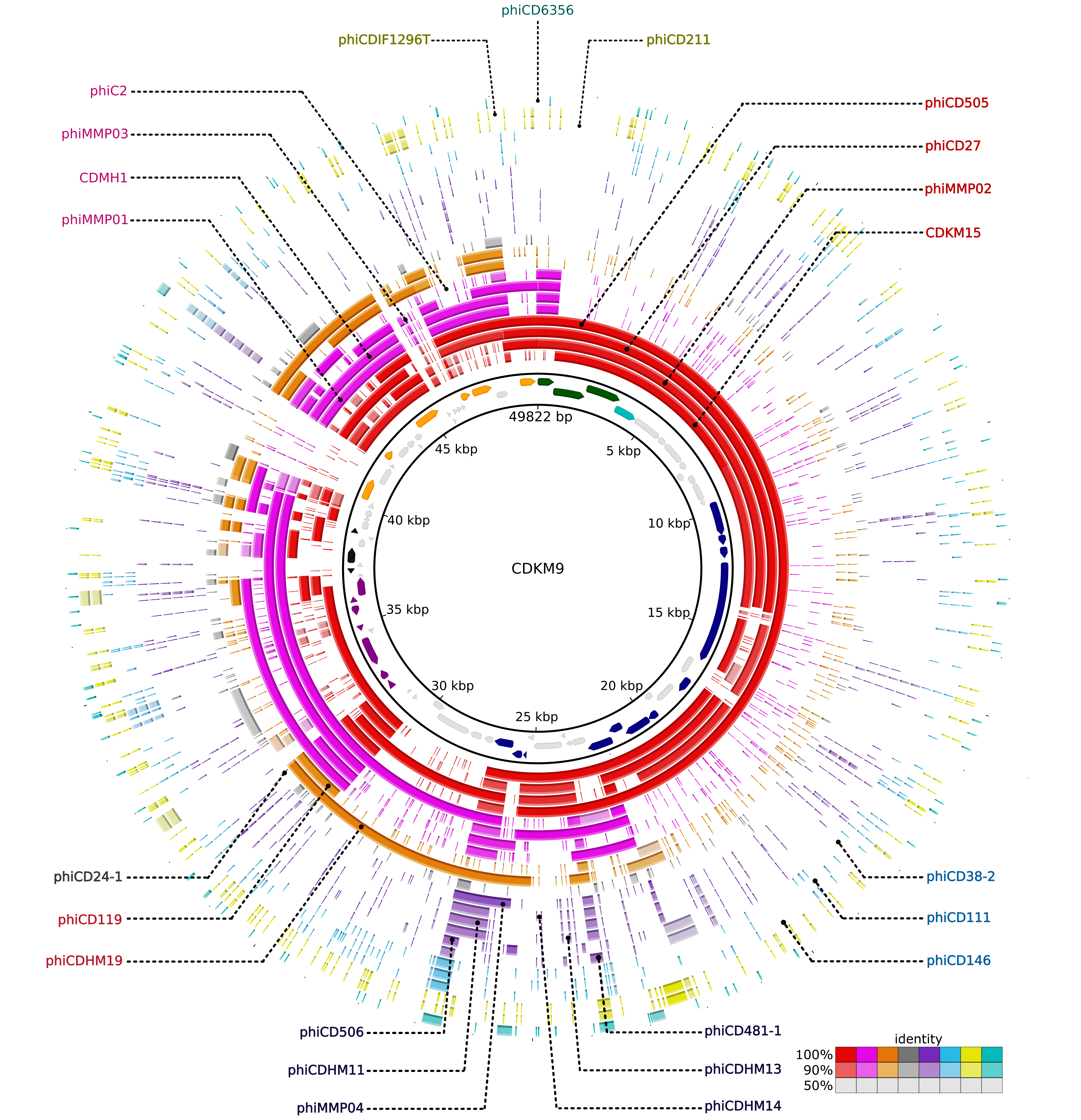


**Figure S1.** Whole genome comparison of phage CDKM9 and other *Clostridium difficile* phages. The local similarity of each phage is calculated based on BLASTn high scoring pairs and plotted against a circular map of the reference genome represented as the inner circle (in this case the genome of CDKM9). Similarity to each of the 23 other C. difficile phages is shown as coloring intensity of consecutive rings. Outer rings are colored consistently with cluster coloring in Figure 6.


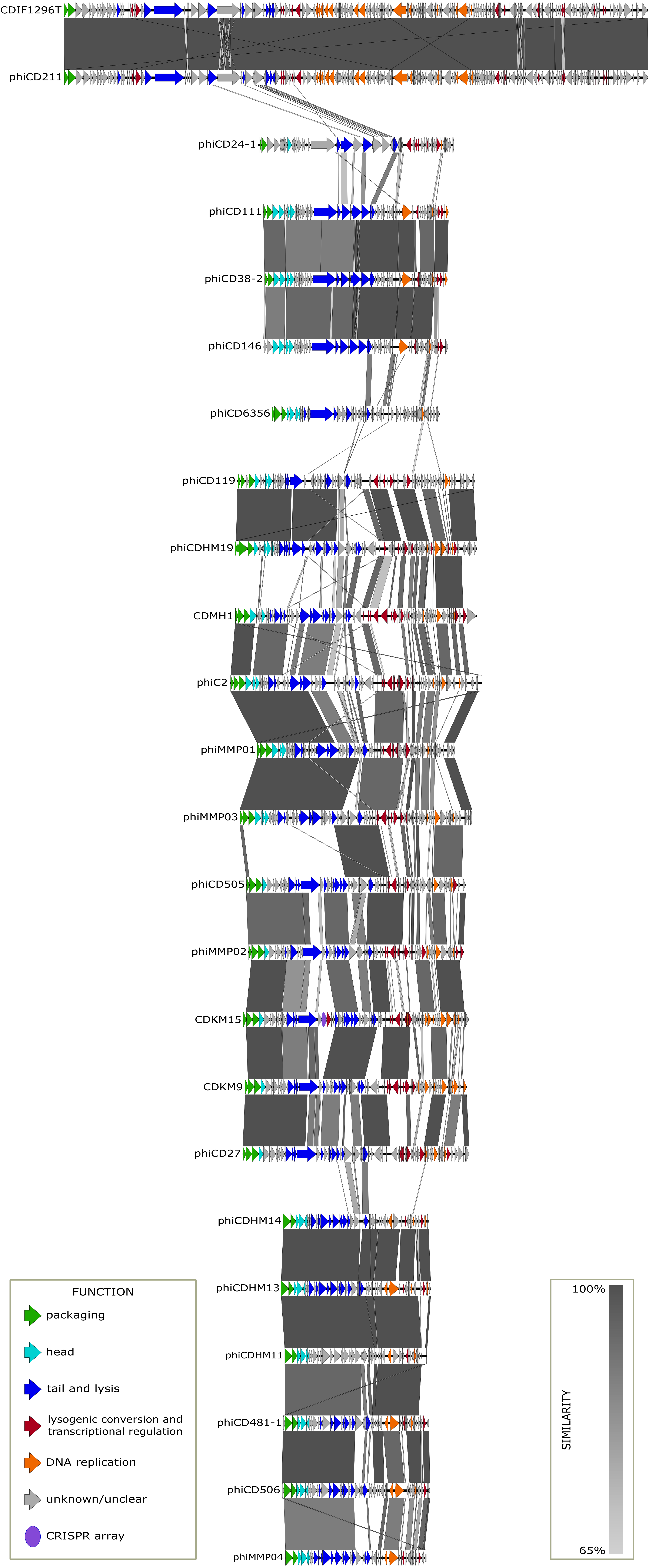


**Figure S2.** Whole genome comparison of all *C. difficile* phages. The linear comparison was generated using EasyFig (BLASTn as the alignment algorithm other settings default). Predicted CDSs are marked with arrows and colors indicate functional modules: head packaging (green); head (aquamarine); tail and lysis (blue); lysogenic conversion and transcriptional control (purple) and DNA replication (orange). CDSs with no function assigned are light grey. The CRISPR array is marked with a mauve oval. Shaded blocks connect regions with similarity ranging from 60% (light grey) to 100% (dark grey). In most cases, function was inferred from existing annotations. In few dubious cases, proteins were re-annotated as described above (see Materials and Methods Section 2.4. “Phage genome sequencing”). Details concerning functional annotation are available in Table S4.

**Table S1.** Bacterial strains used in this study.

**Table S2.** Detailed results of the genome assembly. Sheets list methods as well as resulting contigs/scaffolds. To assemble CDKM9, pairs from the original library were randomly sampled to achieve depth ~300 (with Daniel C. Jones “fastq-sample” script avalaible at [[1](#_ENREF_1)]). Read mapping was performed with the Geneious algorithm (medium–low setting) using all reads available for the analyzed phage. Scaffolds were mapped to each other using the same algorithm (with additional gaps up to 2000 bp allowed). Regardless of the assembly method, complete genome sequence (with PCR verified assembly ends) was found in 1–2 large contigs/gapless scaffolds. The remaining sequences represent contaminations, sequencing/assembly errors or polymorphic variants of regions already included in the main assembly.

**Table S3.** Genes of novel *C. difficile* phages and their protein products. First two sheets list predicted CDSs, their protein products and sources used for their annotation (BLAST hits and InterProScan domains). The third tab shows the data on putative Shine–Dalgarno sequences used to choose between ambiguous start codons. Models of Shine–Dalgarno sites were calculated using the MEME (as a most significantly overrepresented 6–8 bp long motifs in regions 4–16 bp upstream of each of the phage genes). Occurrences of these motifs were located using FIMO. The workbook contains annotated DNA and protein sequences in gff and genbank format (embedded in the relevant tabs of the xmlx file).

**Table S4.** Sequences and database records used in this study. Accession numbers of all phage sequences used in Figures 4 and 7–9, accession numbers of phage endolydsin genes used in Figure 5 and accession of phage *terL* genes used in Figure 6. Sequences were downloaded from GenBank and RefSeq databases (if the reference sequence was avalaible).

**Table S5.** Host range analysis of the examined phages. Host range analysis was conducted by applying 10 µL of 10^8^ PFU/mL of phage stocks on lawns of bacteria and incubated anaerobically at 37 °C for 24 h. Key: Black
cells = clears; grey cells = turbid clearing; white cells = no clearing. In total, 22 Kurdistan isolates, 55 UK isolates, and one isolate from each Switzerland, France and USA belonging to 20 ribotypes.

**Table S6.** Results of protein cluster analysis and genome comparision. First tab is the matrix showing the number of members from each consecutive protein cluster. Second tab is lists of all members with full description and database references. Third and fourth tabs include similarity matrices resulting from whole genome comparisons of 24 *C. difficile* phages (direct DNA–DNA comparision and translated comparision, respectively).

References

1. Fastq-tools. Available online: http://homes.cs.washington.edu/~dcjones/fastq-tools/ (accessed on
2 November 2016).

© 2016 by the authors. Submitted for possible open access publication under the
terms and conditions of the Creative Commons Attribution (CC-BY) license (http://creativecommons.org/licenses/by/4.0/).
